# Preclinical evaluation of DC-CIK cells as potentially effective immunotherapy model for the treatment of glioblastoma

**DOI:** 10.1038/s41598-024-84284-5

**Published:** 2025-01-03

**Authors:** Annika Simone Lück, Jingjing Pu, Ahmad Melhem, Matthias Schneider, Amit Sharma, Ingo G. H. Schmidt-Wolf, Jarek Maciaczyk

**Affiliations:** 1 Department of Stereotactic and Functional Neurosurgery, University Hospital of Bonn, 53127 Bonn, Germany; 2 Department of Integrated Oncology, Center for Integrated Oncology (CIO), University Hospital of Bonn, 53127 Bonn, Germany; 3 Department of Neurosurgery, University Hospital Bonn, Bonn, Germany; 4 Department of Surgical Sciences, Otago University, Dunedin, New Zealand

**Keywords:** DC-CIK cells, Cytokine-induced killer cells, Immunotherapy, Glioblastoma, Cancer, Immunology, Neurology, Oncology

## Abstract

Despite the favorable effects of immunotherapies in multiple types of cancers, its complete success in CNS malignancies remains challenging. Recently, a successful clinical trial of cytokine-induced killer (CIK) cell immunotherapy in patients with glioblastoma (GBM) has opened a new avenue for adoptive cellular immunotherapies in CNS malignancies. Prompt from these findings, herein, we investigated whether dendritic cells (DC) in combination with cytokine-induced killer cells (DC-CIK) could also provide an alternative and more effective way to improve the efficacy of GBM treatment. The analysis showed that DC-CIK cells exerted a significant cytotoxic effect on the glioblastoma cell lines, especially with the phenotype of stem-like cells (GSCs). In addition, the increased specific lysis of these cells subsequent to DC-CIK co-culture was confirmed with confocal fluorescence microscope. The direct interactions between tumor and effector cells were found to be highly effective in GBM organoids (GBOs). Moreover, a significant increase in apoptosis and elevated levels of IFN-γ (and not TNF-α) secretion were observed as a targeting mechanism of DC-CIK cells against GBM cell models. Overall, we provide important preliminary evidence that DC-CIK cells may have potential in the treatment of CNS malignancies, particularly glioblastoma.

**Supplementary Information:**

The online version contains supplementary material available at 10.1038/s41598-024-84284-5.

## Introduction

Adoptive cellular immunotherapy (ACT) is a form of treatment that uses the cells of patients own immune system to eliminate cancer. ACT types have proven to be very successful in various cancers^1,2^, in particular cytokine-induced killer (CIK) cell immunotherapy, which was successfully used in clinical routine over the last three decades^3,4^. Interestingly, advanced forms of CIK cells therapies such as DC-CIK (dendritic cells combined with CIK cells)^5–7^ and CAR-CIK^8,9^ have demonstrated their successful clinical utility in the cancer therapy.

Strikingly, immunotherapies and other treatment modalities have limited to moderate success in central nervous system (CNS) malignancies like glioblastoma (GBM, previously known as glioblastoma multiforme)^10,11^. GBM (grade IV) is known to affect typically older adults, with diagnosis usually occurring at the age of 65 years, more frequently in men than women^12–14^. A recent study reported that the combination of CIK cell immunotherapy with the standard Stupp protocol (radiotherapy and temozolomide) can potentially prolong both overall survival (OS) and progression-free survival (PFS) in GBM patients without causing significant adverse effects^15,16^. In addition, the results of a Phase III trial using autologous CIK cells also reported improvements in PFS in newly diagnosed GBM patients, whereas OS needed further investigation^17^. This raises the possibility of further improved therapies based on CIK cell immunotherapy. Of note, studies of dendritic cell vaccination (DCV) in GBM patients have also been conducted, confirming feasibility, safety and improved survival^18,19^. Thus, there is a strong rational to test the possibility of combining DC cells with CIK cell immunotherapy in GBM patients.

Considering this, we investigated the suitability of dendritic cell-modified CIK (DC-CIK) therapy using GBM cellular models, including glioma stem like cells (GSCs) and glioblastoma organoids (GBOs). Cytokines such as IFN-γ and TNF-α are key mediators of antitumor immunity, with IFN-γ known to enhance the antigen presentation and cytotoxicity of immune cells, while TNF-α plays a pivotal role in inducing apoptosis in GBM cells and modulating the tumor microenvironment^20–22^. The WNT/β-catenin signaling pathway, frequently dysregulated in glioblastoma, has been implicated in maintaining glioma stem-like cell populations, promoting tumor progression, and driving resistance to therapy^23,24^. To undermine the effectiveness and molecular mechanisms related to DC-CIK cells and GBM cells, we investigated several parameters such as release of IFN-γ, TNF-α and expression level of WNT/beta-catenin pathway.

## Materials and methods

### Cell lines and cell culture

#### Target cells

Three primary glioblastoma cell lines (G35, 84 and BTSC233) were cultured at 37 °C, 5% CO2. All cell lines are IDH wildtype. Glioblastoma cell lines G35 and 84 were generously provided Dr. Matthias Schneider (University Hospital Bonn, Germany) and Maria Stella Carro (University of Freiburg, Germany). Briefly G35 and 84 were cultured in DMEM (Gibco, Grand Island, NY, USA), supplemented with FBS (Gibco, Grand Island, NY, USA) and Penicillin/ Streptomycin (Gibco, Grand Island, NY, USA) to initiate differentiation and adherence^25^. Primary glioblastoma stem like cell line BTSC233 was kindly provided by M.S. Carro, Freiburg University, Freiburg im Breisgau, Germany and cultured as previously described^26^. Briefly spheroid cultured BTSC233 was cultured in Neurobasal medium supplemented with B27 (Gibco, Grand Island, NY, USA) and N2 (Gibco, Grand Island, NY, USA), FGF (R&D Systems, Minneapolis, USA), EGF (R&D Systems, Minneapolis, USA), LIF (Miltenyi Biotec, Bergisch Gladbach, Germany), Heparin (Sigma-Aldrich, Saint Louis, USA) and Glutamax (Gibco, Grand Island, NY, USA). Adherent differentiated cultured BTSC233 cells were induced as noted above for cell lines G35 and 84. Glioblastoma Organoids (GBO) were cultured as described before^27^. Briefly, Organoids were cultured in a 24 wells-plate on an orbital shaker at 37 °C, 5% CO2 in GBO-Medium, which consists of 235 ml of DMEM: F12 medium, 235 ml of Neurobasal medium, 5 ml of MEM-NEAAs solution (100×), 5 ml of GlutaMAX supplement (100×), 5 ml of penicillin-streptomycin (100×), 5 ml of N2 supplement (100×), 10 ml of B27 minus vitamin A supplement (50×) and 125 µl of human recombinant insulin. The needed amount of 2-mercaptoethanol (1,000×) was added to an aliquot of medium immediately before changing the GBO medium. Plasticware optimized for adherent cells was used for differentiated adherent cells and plasticware optimized for suspension cells was utilized for spheroid stem cells and GBOs.

#### Effector cells

Isolation of peripheral blood mononuclear cells (PBMCs) from buffy coat (University Hospital Bonn) of healthy donors was performed as described by Schmidt-Wolf et al.^28^. PBMCs were seeded in a 6-well-plate at 25 × 10^6^ cells per well in 4 mL DC- medium (RPMI-1640 medium supplemented with 10% sterile filtered serum from individual donor and 100 U/ml penicillin, 100 mg/ml streptomycin). After 3 h of incubation at 37 °C the supernatant containing non-adherent cells was replaced with fresh DC-medium supplemented with 1000U/ml GM-CSF and 1000 U/ml IL-4 (ImmunoTools, Friesoythe, Germany). DC-Medium with cytokines was exchanged every 2–3 days. Tumor lysate for DC pulsing was extracted from each cell lines via five freeze-thaw cycles at 1 × 10^7^ cells / mL PBS. To determine the protein concentration of the supernatant Pierce BCA Protein-Assay kit (Thermo Scientific, USA) was utilized. Aliquoted tumor lysate was stored at − 80 °C. On day 6 DC were getting pulsed with 100ug tumor lysate/ mL DC-Medium for 48 h. DC-Medium was refreshed after 48 h, supplemented with 1000 U/ml TNF-a (ImmunoTools, Friesoythe, Germany) and cytokines as earlier described for 24 h.

Simultaneously Cytokine-induced Killer (CIK) cells were generated from the same donor and cultured in CIK- medium (RPMI-1640 medium supplemented with 10% FBS, 2.5% HEPES and 1% penicillin/streptomycin). On day 9 CIK cells and DC cells were co-cultured at a ratio 1:5 DC: CIK in CIK-medium supplemented with IL-2 as outlined previously^5^. For phenotypic identification by flow cytometry, DC-CIK cells were harvested on day 14. Cells were stained with FITC-CD3, PE-CD56, and V450-CD8 and corresponding isotype antibodies.

### Co-culturing effector and target cells

Differentiated cells from cell line G35 and 84 were cultured in 48-well plates at 3 × 10^4^ cells per well in co-culture media (50% CIK-medium, 50% target cell medium). After at least 1 h of incubation at 37 °C cells did adhere to the wells and DC-CIK cells (or CIK cells) were added to the wells in effector-to-target (E: T) ratios 10:1, 20:1 (and 40:1) for 24 h and 48 h.

For ELISA assays 1 × 10^4^ glioblastoma cells were incubated with E: T 20:1 for 16 h in a 96-well-plate. BTSC233 was co-cultured with DC-CIK as spheroid stem cells and differentiated adherent cells in various E: T ratios. Plasticware optimized for suspension cells was used for spheroid BTSC233 cells using Neurobasal medium with supplements as described above. Co-culture for differentiated cells was performed as described for G35 and 84.

GBOs and DC-CIK were co-cultured in 50% CIK-Medium and 50% GBO-Medium on an orbital shaker with optimized E: T ratios depending on the experiment.

Co-culturing with transwell system (TC-insert, pore size 0.4 μm, Sarstedt AG & Co. KG, Nümbrecht, Germany) was performed at E: T ratio 10:1 with BTSC233 and GBOs for up to 72 h.

### Cytotoxicity assay

Target cells were incubated 20 min at 37 °C using 5 µM fluorescent CFSE (Thermo Fisher Scientific, Eugene, USA) cell dye in 1 × 10^6^ cells/mL DMEM. CFSE-labeled target cells were washed with culture medium afterwards for two times. Target cells were seeded first into the plates allowing them to adhere or form spheroids. Later effector cells have been added in different effector-to-target (E: T) ratios for 24 and 48 h using triplicates for each ratio and control. After coculturing at 37 °C time all samples have been harvested. Cell viability was measured immediately by flow cytometry, using a FACS Canto II Flow Cytometer (BD Biosciences, Heidelberg, Germany) as outlined previously^29^. In brief cell viability dye Hoechst 33,258 (Cayman Chemical, Hamburg, Germany) was added to each sample and 10.000 CFSE-positive cells were analyzed per replicate. Specific lysis was calculated as following:

$${\text{Specific}}\:{\text{lysis}}\left( \% \right) = \left( {\frac{{{\text{CT}} - {\text{TE}}}}{{{\text{CT}}}}} \right) \times 100$$

CT: absolute number of live CFSE labeled tumor cells in control tubes (target cells alone); TE: absolute number of live CFSE labeled tumor cells in test tubes (target cells and effector cells).

### Apoptosis assay

Labelling target cells was conducted using CellTrace Violet (Thermo Fisher Scientific, Waltham, MA, USA) dye according to the manufacturer’s recommendations. Effector cells were added in E: T ratios 10:1 and 20:1 for 24 h. According to FITC Annexin V Apoptosis Detection Kit with 7-AAD (BioLegend, San Diego, USA) samples got stained after coculturing and analyzed. Cells got collected, washed with PBS and then resuspended in 100 µL Annexin V binding buffer per sample. After adding 5 µL FITC Annexin V and 5 µL 7-AAD into each tube, samples were incubated on ice for 20 min. Subsequently, 400 µL Annexin V binding buffer were added per sample and measured by flow cytometry. Apoptosis assay was repeated for 3 different buffy coat donors on cell lines G35 and 84 using triplicates for each E: T ratio and controls for each donor.

### ELISA assay for IFN-γ and TNF-α release

First, DC-CIK cells and attached glioblastoma cell lines G35 and 84 were co-cultured for 16 h at E: T ratio 20:1. For each experiment triplicates have been used and DC-CIK cells alone as a control group. Media and glioblastoma cells were cultured alone to proof the measured IFN-γ and TNF-α release is just contributed from DC-CIK cells. After the co-culture period 100 µL supernatant was collected, centrifuged at 10,000 rpm for 5 min and stored at -80 °C if needed. Cytokine release was quantified by IFN-γ ELISA kit (Invitrogen, Camarillo, CA, USA) and TNF-α ELISA kit (Invitrogen, Camarillo, CA, USA) according to manufacturer’s protocol. For BTSC233 spheroid cultured stem cells E: T ratio needed to be adjusted to 10:1, followed procedures remained the same. GBOs were co-cultured at E + T 1:1 for direct contact co-culture and 10:1 with transwell system. Supernatant was collected after 24 h, 48 h and 72 h from each group and processed as explained above.

### GBO co-culture and fluorescent immunohistology

GBOs and DC-CIK cells were co-cultured with direct cell-cell contact at a ratio of E: T 1:1 and 10:1 with transwell system as mentioned for the ELISA Assay. Viable cell number was quantified by GBOs’ diameter^27^ and similar sized GBOs were used. GBOs were imaged using the Lionheart FX automated microscope at 24 h, 48 h and 72 h to measure the organoids size and evaluate the shape as described before^30^. For fluorescence microscopy using a confocal microscope, GBOs were co-cultured with CIK cells for 48 h. After coculturing CIK cells and GBOs with direct contact at E: T 1:5 GBOs were washed with 4 ml PBS, and fixed in 4 ml 4% (vol/vol) formaldehyde solution for 30 min at RT. The GBOs were then cryoprotected by incubating them in 4 ml 30% (wt/vol) sucrose solution at 4 °C overnight, before embedding them in plastic cryomolds in tissue freezing medium (General Data, cat. no. 1518313) and snap freezing them in isopropanol cooled by dry ice. The cryomolds were stored at -80 °C until the day of sectioning, when they were equilibrated to -20 °C and sectioned into 25 μm sections using a tissue cryostat. The sections were mounted on a charged microscopic slide and dried on a hotplate at 55 °C for 30 min. Staining of the sections for Immunofluorescence was performed as described previously^27^. To assess cell death, diluted primary antibody for cleaved caspase 3 (Rabbit anti-cleaved caspase-3 polyclonal antibody, Cell Signaling Technology, Massachusetts, USA) was incubated with the samples. To differentiate between tumor cells and CIK cells we used T cell marker anti-CD3 (Mouse anti-CD3 monoclonal antibody, BioLegend, San Diego, USA) to stain CIK cells. Hoechst 33,342 (Life Technologies, California, USA) was utilized to stain all nuclei. All confocal images of fluorescent immunohistology for CD3, CC3 and Hoechst 33,342 on controls and co-cultured samples were taken with Visiscope CSU-W1 with VS-homogenizer spinning disk microscope.

### RT qPCR detection of genes related to Wnt/beta-catenin pathway RNA expression level

First target cells were labeled using CFSE as outlined above. DC-CIK cells were added at E: T ratio 1:1 for 24 h. After coculturing time cells were collected, centrifuged and resuspended in warmed co-culture-medium. Then unlabeled effector cells were sorted out using cell sorter Aria III (BD Biosciences, Heidelberg, Germany). CFSE labeled effector cells were collected and washed with PBS afterward, pellets were frozen at -80 °C for storage.

RNA extraction and quantitative polymerase chain reaction. RNA isolation of GBM cell line (G35, 84 and BTSC233) samples was performed with RNeasy Plus Mini Kit (QIAGEN, Germany, Cat. No: 74136). Complementary DNA was synthesized by reverse transcription using hifiscript Kit (Invitrogen, USA). Quantitative polymerase chain reaction was performed on genes related to Wnt/beta-catenin signaling pathway (Wnt2b, Wnt3, Wnt3a, Wnt5a, Wnt7b, Wnt11, FZD2, FZD6, FZD7, β-catenin, APC, GSK3β, Axin1, Axin2 and Cyclin D1) using P PowerTrack™ SYBR Green Mastermix (Thermo Fisher Scientific, Cat. No: A46109). β-actin was selected as the internal reference gene. The primer sequence is as follows (Supplementary Table 1). The Δ–Δ Ct (2–∆∆Ct) approach was used to measure the relative expression levels of target genes, which were standardized against β-actin mRNA levels.

### Statistical analysis

FACS data were analyzed using FlowJo V10.4 software (LLC, Ashland, Oregon, USA). GraphPad Prism (version 8.0) was used for statistical analysis. All presented experimental data are means ± standard deviation. Statistical significances are illustrated as following: * meaning *p* < 0.05, ** stands for *p* < 0.01, *** represents *p* < 0.001, and **** symbolizes *p* < 0.0001. *P* < 0.05 was regarded as statistically significant.

## Results

### Phenotypic identification of CIK cells and in vitro cytotoxic effect of DC-CIK on glioblastoma cell lines

We first characterized the phenotype of CIK cells, confirming that NK-T (CIK) (CD3^+^CD56^+^) cells accounted for 12.5% of PBMCs and CD8^+^ NK-T cells comprised 57.2% of the population (as shown in Supplementary Fig. 1A) thus harboring obvious cytotoxic effects. DC-CIK cells were then co-cultured with glioblastoma cell lines using G35 and 84 as differentiated attached cells and BTSC233 as spheroid stem cells. For each cell line, three different buffy coat donors with different effector-to-target (E: T) ratios were used, ranging from 1:1 to 40:1.

**Fig. 1:**
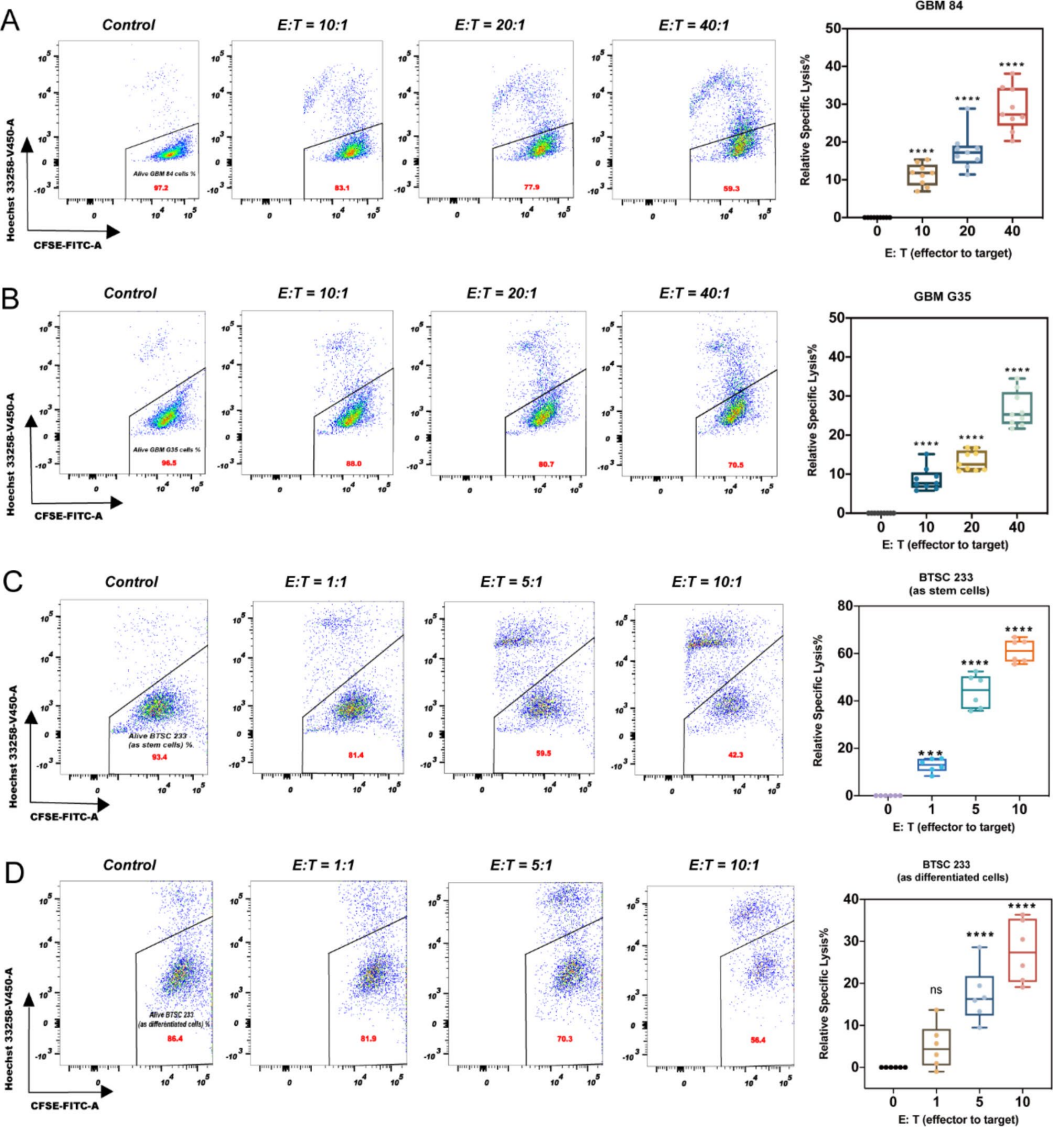
Cytotoxic effect of DC-CIK cells on primary glioblastoma cell lines (84, G35 and BTSC233). (**A**) 84 cells were cultured for 24 h at different effector: target (E: T) ratios of 10:1, 20:1 and 40:1. (**B**) G35 cells were cultured for 24 h at different E: T ratios of 10:1, 20:1 and 40:1. (**C**) BTSC233 (as stem cells) were cultured for 24 h at different E: T ratios of 1:1, 5:1 and 10:1. (**D**) BTSC233 (as differentiated cells) were cultured for 24 h at different E: T ratios of 1:1, 5:1 and 10:1. The cytotoxic effect of DC-CIK cells was measured by flow cytometry. The results represent data from three separate experiments and are presented as mean ± SD. Significance levels were determined using two-way ANOVA with Bonferroni’s post-hoc test (*P < 0.05, ** *P* < 0.01, *** *P* < 0.001, **** *P* < 0.0001).

For G35 and 84, E: T ratios of 10:1, 20:1, and 40:1 significantly reduced the in vitro viability of glioblastoma cells (*P* < 0.0001). The increase of the effector ratio led to an increase in the relative specific lysis of glioblastoma cells in both cell lines (Fig. 1A and B). In 84, we found that 48 h of co-culture showed more relative specific lysis than 24 h (20:1; *P* < 0.0001). Co-culturing G35 cells for 48 h with DC-CIK cells also resulted in an increased cytotoxic effect, albeit less strongly (20:1; *P* < 0.05) (Supplementary Fig. 1B).

**Fig. 2:**
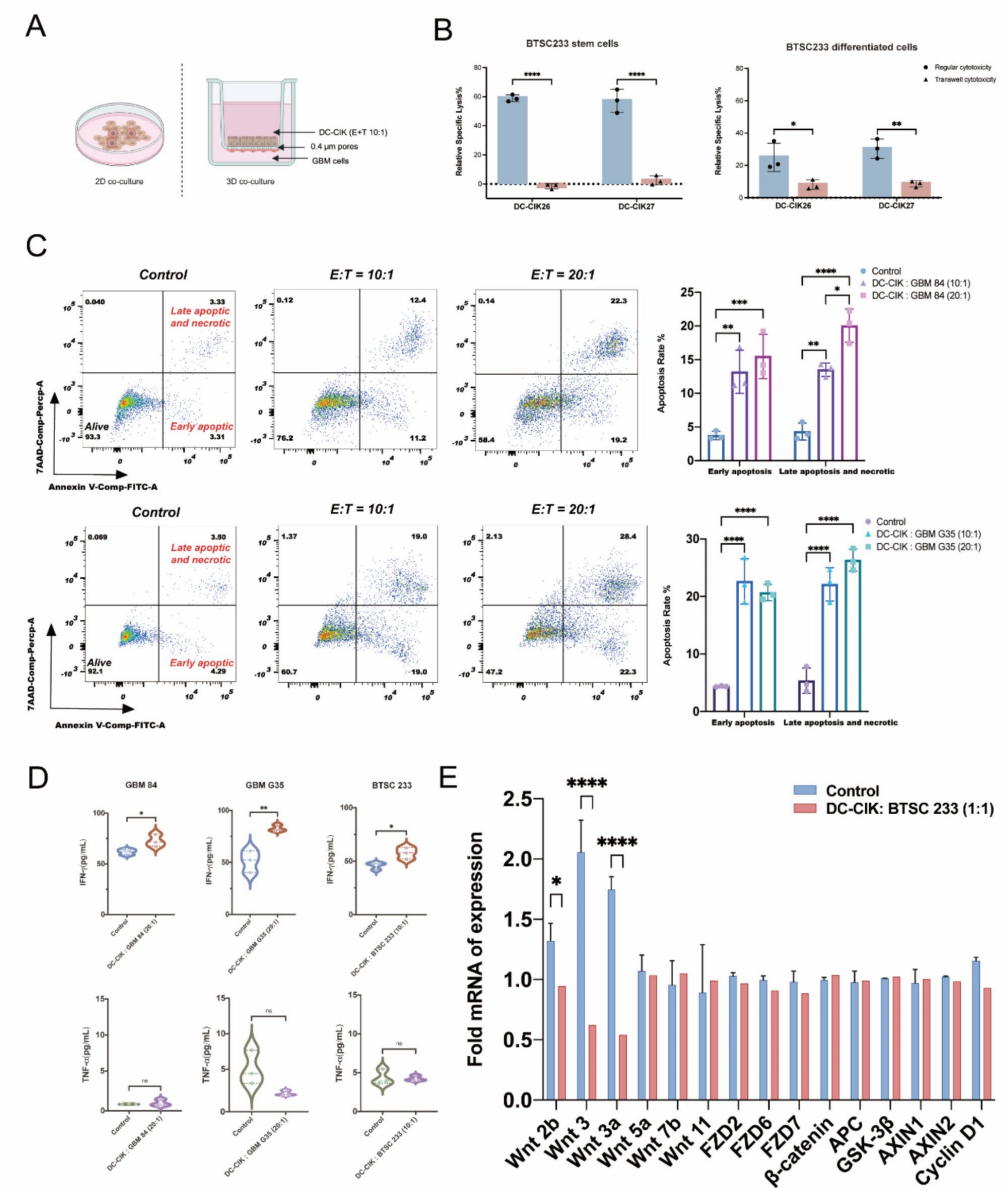
(**A**) Schematic of 2D single-cell and 3D spheroid models for co-culturing DC-CIK and GBM cells in a transwell system. (**B**) BTSC233 (stem cells and differentiated cells) were cultured at a 10:1 effector: target ratio for 24 h comparing regular cytotoxicity and transwell cytotoxicity. The cytotoxic effect of DC-CIK cells was measured by flow cytometry. (**C**) DC-CIK cells on the apoptosis of GBM cell lines (84 and G35) by the Flow Cytometry assay. Flow cytometry figure of changes in the proportion of early apoptosis cells and late apoptosis or necrosis cells. Cells were stained with FITC Annexin V and Percp 7AAD. The result is one of the representative data. (**D**) IFN-γ and TNF-α secretion in DC-CIK cells targeting GBM cell lines 84, G35, and BTSC233. (**E**) Relative mRNA expression changes of Wnt/beta-catenin pathway-related genes on BTSC233 (spheroid) cell line after coculturing with DC-CIK cells (E: T ratio 1:1) for 24 h. The results represent data from three separate experiments and are presented as mean ± SD. Significance levels were determined using two-way ANOVA with Bonferroni’s post-hoc test (*P < 0.05, ** *P* < 0.01, *** *P* < 0.001, **** *P* < 0.0001).

**Fig. 3:**
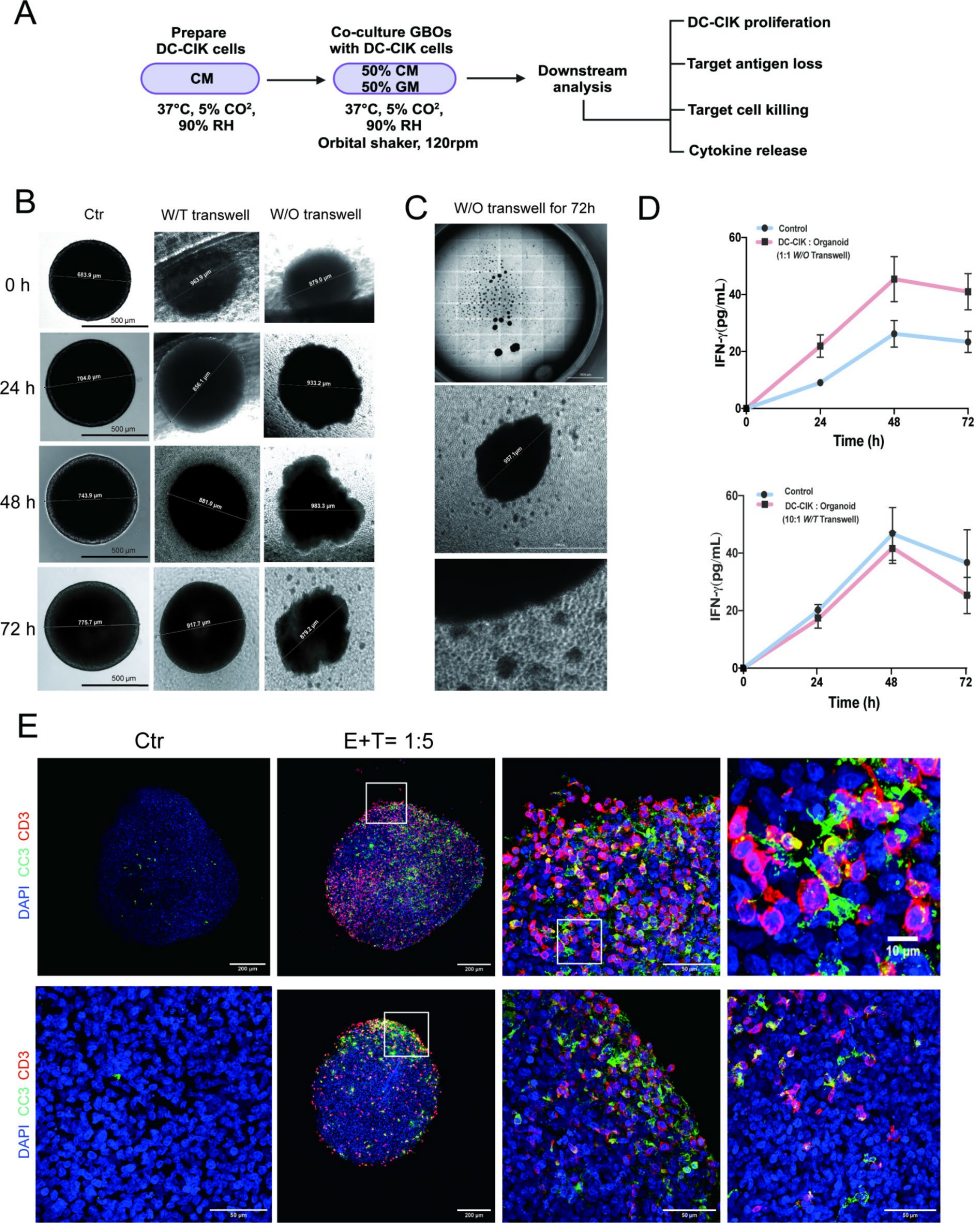
(**A**) Diagram outlining the steps to prepare and co-culture DC-CIK cells with GBOs with downstream analyses. The medium and culture conditions are indicated below each step. CM, CIK cell medium; GM, GBO medium; RH, relative humidity. (**B**) Phase contrast images of individual GBOs at 0, 24, 48, and 72 h, co-cultured with DC-CIK cells with or without a transwell system, and a control group of GBOs alone. Representative images are based on the analysis of 10 GBOs. Scale bar, 500 μm. (**C**) Sample phase contrast images of the edge of GBOs when transwell co-cultured with DC-CIK cells for 72 h (E: T 10:1 W/T transwell E: T 1:1 W/O transwell). Representative images are based on the analysis of 10 GBOs. (**D**) Quantification of IFN-γ in media from co-cultures with DC-CIK cells, with or without a transwell system, was performed using ELISA at 0, 24, 48, and 72 h. Values represent mean ± s.e.m. (*n* = 3 GBOs per sample per timepoint). (**E**) Sample confocal images of fluorescent immunohistology for cleaved caspase 3 (CC3) and cluster of differentiation 3 (CD3) in GBOs after co-culture with either CIK cells for 48 h (E: T 1:5), showing increased apoptosis when co-cultured with CIK cells, with higher-magnification images of boxed regions highlighting apoptotic cells near CIK cells at the GBO periphery and core. Representative images are based on the analysis of five GBOs. Images were taken using Zen 2 Blue software and were processed using Illustrator CC, ImageJ, Imaris and/or Photoshop CC software.

Notably, DC-CIK cells exhibited a significant effect on the BTSC233 cell line at lower E: T ratios, similar to those observed with G35 and 84. BTSC233 was also co-cultured with DC-CIK cells from three healthy donors for 24 h. When comparing stem cells and differentiated cells, an E: T ratio of 1:1 significantly decreased the viability of BTSC233 stem cells (1:1; *P* < 0.001), while the viability of BTSC233 differentiated cells was not significantly affected (1:1; ns) (Fig. 1C and D). Notably, under bright field microscope we observed higher specific lysis in the form of clusters in BTSC233 (Supplementary Fig. 1C). DC-CIK cells appear to perform better in 3D models as they form clusters around tumor cells. Furthermore, we investigated whether DC-CIK cells have a cytotoxic effect on BTSC233 cells without direct cell-to-cell contact by using a transwell-system (Fig. 2A, 3). In contrast to direct effector and tumor interactions, transwell systems significantly reduced specific lysis for BTSC233 cells (2 donors, triplicate each) (Fig. 2B). Overall, DC-CIK showed the highest cytotoxic effect on BTSC233 indicating that the targeting cells cultured in a 3D spheroid model (rather than a 2D single-cell model) may be an effective way to activate cytotoxic mechanisms for their function.

### Apoptotic effect, IFN-γ and TNF α secretion of DC-CIK cells on glioblastoma cell lines

After co-culturing for 24 h, early apoptotic cells, as well as cells undergoing late apoptosis or necrosis, in the 84 and G35 cell lines were measured using 7-AAD and Annexin V at E: T ratios of 10:1 and 20:1 for 24 h. To mention, a significant increase in both levels of apoptosis was observed in the DC-CIK co-culture with G35 and 84. Higher effector cell ratios led to an increased number of target cells in the stage of late apoptosis and necrosis in cell line 84 (*P* < 0.05) (Fig. 2C). Additionally, we quantified the secretion of cytokines, specifically IFN-γ and TNF-α, using a sandwich ELISA. Since, DC-CIK cells also release cytokines even in the absence of contact with tumor cells, their baseline levels were compared to cytokine levels in co-culture supernatants. After evaluating various time points, a 16-hour co-culture duration was found to be optimal for cytokine release. IFN-γ secretion increased significantly in co-cultures in all three cell lines (G35, *P* < 0.01; BTSC233, *P* < 0.05) (Fig. 2D). In contrast, TNF-α levels remained similar to the basal levels in all cell lines despite the initial low basal secretion (Fig. 2D). This suggests that IFN-γ plays a crucial role in the killing mechanisms of DC-CIK cells against GBM cell lines, mediated through the apoptosis of target cells. Furthermore, we found that coculturing GBM Organoids and DC-CIK with direct contact leads to an increased IFN-γ release, whereas co-culture with transwell does not have this effect. Notably, the size of Organoid in transwell does not shrink during co-culture (Fig. 3B).

### mRNA expression changes of Wnt/beta-catenin pathway-related genes

Since the Wnt/beta-catenin pathway plays an important role in the onset and development of GBM^31^, this pathway is considered extremely important when immunotherapeutic interventions are envisioned.

Thus, we next investigated the changes in the expression of genes related to the Wnt/beta-catenin signaling pathway following exposure of DC-CIK cells towards GBM cell lines using qRT-PCR. Interestingly, we found that the expression of WNT 2b (*P* < 0.05), WNT3 (*P* < 0.001) and WNT3a (*P* < 0.001) were reduced after co-culture in BTSC233 (Fig. 2E). However, there were no effects on Wnt/beta-catenin for DC-CIK cells on G35 and 84 (Supplementary Fig. 1D). This suggests that DC-CIK cells have limited ability to alter Wnt/beta-catenin pathway. Thus, it offers a possibility of safe testing possible synergistic effects of DC-CIK cell therapy with Wnt/beta-catenin inhibitors and, even more broadly, with immune checkpoint inhibitors applicable for GBM treatment.

### In vitro activity of CIK and DC-CIK cells against GBM organoids

Like DC-CIK cells, we also compared the cytotoxic effect of CIK cells on G35 and 84 cell lines and found no relative difference between CIK and DC-CIK (Supplementary Fig. 1F). Since the effect of DC-CIK cells were significant in GBM stem like cells (BTSC233), we evaluated the effect of DC-CIK on glioblastoma organoids (GBO), which were generated from patients’ tumors. GBOs were co-cultured for up to 72 h in transwell systems and in direct contact with DC-CIK (Fig. 3A). The supernatant was collected every 24 h to assess IFN-γ secretion at different time points and cells were imaged under a phase contrast microscope. DC-CIK cells in direct contact with the GBOs showed proliferation in the form of clusters surrounding the GBOs, as visualized in phase contrast microscopy (Fig. 3B). After 72 h of co-culture, several clusters were visible around the organoids. The initial E: T ratio at day 0 was considered to be 1:1. Evaluation of the size and density of the cells under phase contrast suggests that the DC-CIK cells have a high proliferation rate. Similar results were found in fluorescence microscopy images of GBO and DC-CIK (E: T 1:1) co-cultured at 0 h and 24 h (Supplementary Fig. 1E). When comparing the natural IFN-γ secretion of DC-CIK cells alone with that of DC-CIK cells in co-culture after 24, 48 and 72 h, we observed higher values in the co-cultured samples at all time points, with a peak value after 48 h (Fig. 3D). This indicates that activation of IFN-γ release is crucial when DC-CIK cells come into direct contact with tumor cells. Samples used for immunofluorescence microscopy were co-cultured at an E: T ratio of 1:5 with CIK cells, not DC-CIK, to ensure that all effector cells were stained with anti-CD3. Confocal images of co-cultured GBOs showed an increase in apoptosis (CC3 immunoreactivity), especially on the surface of GBOs, compared to control GBOs. CD3^+^ cells were observed on both the surface and core of GBOs, mainly near apoptotic cells (Fig. 3E).

Further transwell co-culture (E: T 10:1 with DC-CIK) were performed with the same donor for DC-CIK and GBOs. phase contrast microscopy showed no significant decrease in organoid sizes after 72 h (Fig. 3C). In addition, proliferation of DC-CIK cells and clustering were evident. Notably, the surface and shape of the GBOs appeared to remain the same over the 72 h. Quantification of IFN-γ secretion at 24, 48, and 72 h of co-culture did not reveal higher levels of cytokine release in the co-cultured samples compared to the DC-CIK alone samples. Our data suggest that direct contact between effector and tumor cells is crucial for the activation of DC-CIK and CIK cells, thereby enhancing cytokine release and the killing mechanisms of GBM cells.

## Discussion

Antitumor activity and safety of DC-CIK therapy has been demonstrated in studies in a variety of malignancies^32–34^.It has also been emphasized that DC-CIK in combination with chemotherapy has greater clinical efficacy than chemotherapy or DC-CIK treatment alone^35^. Based on the recent success of CIK cell immunotherapy clinical trials^16,17^ and the successful implementation of dendritic cell vaccination (DCV) trials^36^, we aimed to investigate the suitability of DC cells in combination with CIK cell immunotherapy (DC-CIK) for GBM patients.

To determine this, we first cultured DC-CIK cells with GBM cell lines G35, 84 (differentiated adherent cells) and BTSC233 (spheroidal stem-like cells) at different E: T ratios ranging from 1:1 to 40:1. We found that increasing the effector ratio led to an increase in the relative specific lysis of GBM cells. Importantly, an E: T ratio of 1:1 significantly decreased the viability of BTSC233 stem cells (1:1; *P* < 0.001), and we also observed higher specific lysis using confocal fluorescence microscope on GBOs. This suggests that DC-CIK cells performed better in the 3D spheroid model (and not in the 2D cell leyer model), as they can form clusters around tumor cells. In other words, direct cell interactions between tumor and effector cells seems to be an important prerequisite for the activation of cytotoxic mechanisms. Next, we observed a significant increase in apoptosis (early and late) in DC-CIK co-culture with G35 and 84. Notably, IFN-γ secretion in the co-cultures increased significantly in all three cell lines, while TNF-α levels remained unchanged. This indicates that IFN-γ plays an important role in the killing mechanisms of DC-CIK cells against GBM cell lines. However, further experiments are needed to substantiate the individual effects of IFN-γ and other factors besides TNF- α that may have synergistic effects on the killing mechanisms of DC-CIKs. In addition, we investigated whether the Wnt/beta-catenin signaling pathway, which plays an important role in the onset and development of GBM^37–39^, was altered by DC-CIK. Of interest, we observed alterations in the expression few components (WNT 2b, WNT3 and WNT3a) of this pathway, primarily in BTSC233. This suggests that DC-CIK cells have a limited ability to alter the Wnt/beta-catenin signaling pathway. Testing the synergistic action of DC-CIK with Wnt/beta-catenin inhibitors in GBM cells may therefore contribute to further insights.

Besides, we investigated the ability of DC-CIK cells and CIK cells to target glioblastoma organoids (GBO) directly (cell-to-cell contact) and indirectly (with the transwell system). DC-CIK cells in direct contact with the GBOs showed proliferation in the form of clusters surrounding the GBOs, as visualized in phase contrast microscopy. While no significant decrease in organoid size and IFN-γ secretion was observed in transwell co-culture with the same donor for DC-CIK and GBOs. Therefore, our data suggest that direct contact between effector and tumor cells is crucial for activating DC-CIK and CIK cells, thereby enhancing cytokine release and the killing mechanisms previously mentioned in GBM cell lines. Importantly, we compared the natural IFN-γ secretion of DC-CIK cells alone with that of DC-CIK cells in direct co-culture with GBOs and observed elevated levels of IFN-γ.

Overall, our data suggest that direct contact between effector and tumor cells is critical for the activation of DC-CIK and CIK cells, thereby enhancing cytokine release and killing mechanisms for GBM cells. DC-CIK cells are primarily more effective in combating GBM cells through apoptosis mediated in part by increased IFN-γ levels. In summary, it provides an important preliminary evidence that DC-CIK cells may have potential in the treatment of CNS malignancies, particularly glioblastoma, further in vivo experiments specifically focused on the therapeutic utility of DC-CIK cells in temozolomide-resistant GBM can help to advance this perspective.

## Electronic supplementary material

Below is the link to the electronic supplementary material.

Supplementary Material 1.

Supplementary Material 2.

## Data Availability

The authors declare that all the data supporting the findings of this study are contained within the paper or are available upon request from the corresponding author.
