# Supplementary material for: Preclinical evaluation of DC-CIK cells as potentially effective immunotherapy model for the treatment of glioblastoma
Source: Sci Rep. 2025 Jan 3;15:734. doi: 10.1038/s41598-024-84284-5 (PMC11698714; doi:10.1038/s41598-024-84284-5)
Supplement: Supplementary file 1 — Supplementary Material 1. [file 41598_2024_84284_MOESM1_ESM.docx]

| **Primer name** | **Forward sequence** | **Reverse sequence** |
| --- | --- | --- |
| Wnt2b | 5′-GCCGTGTCATGCTCAGAA-3′ | 5′-GTGGACTACCCCTGCTGATG-3′ |
| Wnt3 | 5′-CTCGCTGGCTACCCAATTT-3′ | 5′-GCCCAGAGATGTGTACTGCTG-3′ |
| Wnt3a | 5′-CATGAACCGCCACAACAAC-3′ | 5′-TGGCACTTGCACTTGAGGT-3′ |
| Wnt5a | 5′-ATTGTACTGCAGGTGTACCTTAAAAC-3′ | 5′-CCCCCTTATAAATGCAACTGTTC-3′ |
| Wnt7b | 5′-CGCCTCATGAACCTGCATA-3′ | 5′-GCTGCATCCGGTCCTCTA-3′ |
| Wnt11 | 5′-TGTGCTATGGCATCAAGTGG-3′ | 5′-CAGTGTTGCGTCTGGTTCAG-3′ |
| FZD2 | 5′-GGTGTCGGTGGCCTACAT-3′ | 5′-GAGAAGCGCTCGTTGCAC-3′ |
| FZD6 | 5′-TGGGTTGGAAGCAAAAAGAC-3′ | 5′-TCTTCGACTTTCACTGATTGGA-3′ |
| FZD7 | 5′-GCCAGCTTGTGCCTAATAGAA-3′ | 5′-AGCCGGGAGAAACTCACAG-3′ |
| β-catenin | 5′-CTTACACCCACCATCCCACT-3′ | 5′-CCTCCACAAATTGCTGCTGT-3′ |
| APC | 5′-GCCCCTGACCAAAAAGGAAC-3′ | 5′-TGGCAGCAACAGTCCCACTA-3′ |
| GSK3β | 5′-CAAGCCAAACTTTGTGACTCAG-3′ | 5′-TATCAGGATCCAGCAAGAGGTT-3′ |
| Axin1 | 5′-AGCCGTGTCGGACATGGA-3′ | 5′-AAGTAGTACGCCACAACGATGCT-3′ |
| Axin2 | 5′-TGTGAGGTCCACGGAAACTG-3′ | 5′-CGTCAGCGCATCACTGGATA-3′ |
| Cyclin D1 | 5′-TCAAATGTGTGCAGAAGGAGGT-3′ | 5′-GACAGGAAGCGGTCCAGGTA-3′ |
| β-actin | 5′-CCAACCGCGAGAAGATGA-3′ | 5′-CCAGAGGCGTACAGGGATAG-3′ |

**Supplementary table 1:** The primer sequence used in the study
