# Supplementary material for: Preclinical evaluation of DC-CIK cells as potentially effective immunotherapy model for the treatment of glioblastoma
Source: Sci Rep. 2025 Jan 3;15:734. doi: 10.1038/s41598-024-84284-5 (PMC11698714; doi:10.1038/s41598-024-84284-5)
Supplement: Supplementary file 2 — Supplementary Material 2. [file 41598_2024_84284_MOESM2_ESM.docx]

**
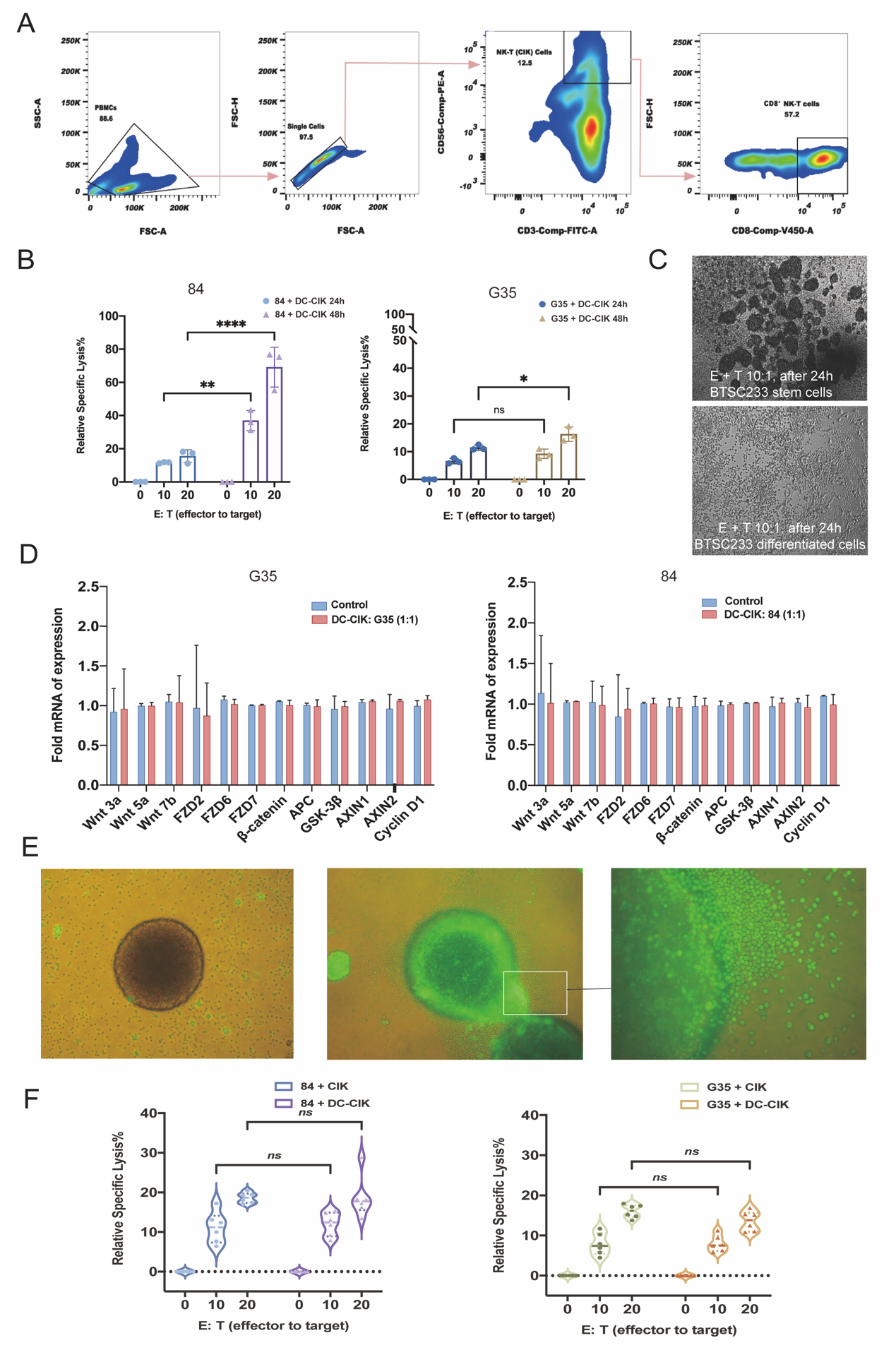
**

**Supplementary figure 1.** In vitro activity of CIK and DC-CIK cells against GBM organoids**. A.** FACS figure of changes in the proportion of CIK cells. CIK cells were prepared from peripheral blood mononuclear cells (PBMCs). Cells were stained with FITC anti-CD3, PE anti-CD56 and V450 anti-CD8 mAbs. Cell surface expression of CD3, CD56 and CD8 was analyzed on live PBMCs by flow cytometry. Data represent one generic buffy coat. **B.** Cytotoxic effect of DC-CIK cells on 84 and G35. GBM cells were cultured for 24 or 48 h at different effector: target ratios of 10:1, 20:1. **C.** Bright field microscopy imaging of BTSC stem cells and differentiated cells after 24 h of culture at an E:T ratio of 10:1. **D.** Relative mRNA expression changes of Wnt/beta-catenin pathway-related genes on G35 and 84 cell lines after coculturing with DC-CIK cells (E:T ratio 1:1) for 24 h. **E.** Fluorescent microscopy images of GBO after 0h and 24h coculturing with DC-CIK (E:T 1:1), DC-CIK stained with CFSE (green). **F.** Cytotoxic effect of CIK and DC-CIK cells on 84 and G35. GBM cells were cultured for 24 h at different effector: target ratios of 10:1, 20:1. The results represent data from three separate experiments and are presented as mean ± SD. Significance levels were determined using two-way ANOVA with Bonferroni’s post-hoc test (*P < 0.05, ** P < 0.01, *** P< 0.001, **** P < 0.0001).
